# Enzymatic browning in relation to permeation of oxygen into the kernel of postharvest areca nut under different storage temperatures

**DOI:** 10.1002/fsn3.2341

**Published:** 2021-05-24

**Authors:** Yonggui Pan, Yuting Guo, Qun Huang, Weimin Zhang, Zhengke Zhang

**Affiliations:** ^1^ College of Food Science and Engineering Hainan University Haikou China; ^2^ Hainan Key Laboratory of Food Nutrition and Functional Food Haikou China

**Keywords:** areca nut, enzymatic oxidation, kernel browning, lignifications, storage temperature

## Abstract

Previous study indicates that kernel of areca nut is susceptible to enzymatic browning caused by phenolic oxidation, which involves the ingression of oxygen into interior tissue. However, the reason for permeation of oxygen into the interior of areca nut and its possible influencing factors (e.g., temperatures) are little known. In the present study, we set three storage temperatures (25, 10, and 5°C) and investigated the effects on kernel browning and related physic‐biochemical and tissue morphological changes. The results showed that the most severe kernel browning was observed in areca nut stored at 25°C, followed by 5°C. Comparatively, a slower browning development was found in areca nut stored at 10°C. More serious kernel browning at 25 and 5°C might be attributed to increased membrane permeability and aggravated tissue damage in view of morphological observations on pericarp, mesocarp, and kernel shell. Higher lignin content and phenylalanine ammonia‐lyase activity were observed in mesocarp of areca nuts stored at 25 and 5°C as compared to 10°C, indicating that mesocarp lignification could facilitate the permeability of oxygen. Furthermore, the data showed that storage at 25 and 5°C induced the higher polyphenol oxidase activity while accelerating the decline in total phenolic content in areca nut kernel, which could contribute to higher occurrence of enzymatic browning reaction compared to that at 10°C. These results suggest that natural senescence at 25°C and severe chilling stress at 5°C could be influencing factors triggering the permeation of oxygen, leading to internal kernel browning in areca nut.

## Graphical abstract

Kernel of areca nuts is highly susceptible to postharvest enzymatic browning. Damage of tissue structure in areca nuts increases the ingress of oxygen. Natural senescence at 25°C and chilling injury at 5°C are inducible factors for triggering the permeation of oxygen, leading to internal kernel browning in areca nut.

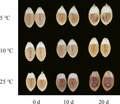

## 1 INTRODUCTION

Areca nut or betel nut is the fruit of the *Areca catechu* tree. It has been estimated that 700 million individuals regularly chew areca nut worldwide (Shafique et al., 2013; Yamada et al., 2013). The fruit contains a variety of beneficial substances, providing human body with a variety of positive effects, such as refreshment, pain relief, and digestive aid (Peng et al., 2015). Although the market is dominated by dry areca nut products, a portion of consumers in China and Southeast Asia countries prefer eating fresh areca fruit (Guo et al., 2020). As areca fruit is harvested from August to December each year in China, the storage of the products is essential to adjust the supply during the off‐season and satisfy the demands of consumers. However, the interior kernel, as a part of edible tissues in areca nut, is susceptible to browning during storage of products, leading to severe quality deterioration and economic loss. Our recent study demonstrated that browning of the areca nut kernel was mainly attributed to enzymatic oxidation of phenolics via catalysis of polyphenol oxidase (PPO) (Guo et al., 2020).

Enzymatic browning does not only require a contact between the PPO present in the cytoplasm and phenolic compounds in cell vacuoles, but also needs the involvement of oxygen (He & Luo, 2007; Mayer, 2006; Zhang et al., 2015). It is generally believed that fruits are vulnerable to enzymatic browning at injured sites when they are bruised, cut, peeled, diseased, or physiologically disordered under abnormal conditions, while less internal browning occurs in healthy fruits due to the presence of natural oxygen barrier system (Jiang & Qu, 2016; Toivonen & Brummell, 2008). For example, the partial pressure of oxygen under the skin of the avocado fruit can drop below 5 kPa due to weak permeability of gas (Valle‐Guadarrama et al., 2002), which effectively avoids the occurrence of internal browning in fruit. However, there are special examples on internal enzymatic browning in a few harvested fruits, such as pears and pineapples. The internal browning in these fruits is known as black heart disease that belongs to a form of physiological disorders (Franck et al., 2007; Saba & Moradi, 2016; Luengwilai et al., 2016).

Huo and Li (1992) observed the “Ya” pear fruit with black heart disease using a scanning electron microscope (SEM), which showed decreased skin wax, increased skin fissure, and increased exposure of skin pore cells to environmental air, thus enhancing the permeability of the fruit skins to gas components. In addition, with the progression of fruit ripening and senescence, adhesion between cells become loose, which facilitates the separation of cells from each other and forms a cell gap (Rao & Ren, 1997). This provides permissive conditions for permeating oxygen into the fruit and results in PPO‐mediated internal enzymatic browning. Unlike internal flesh browning of in pineapple and pear fruits, the internal browning of the areca nut occurs mainly in the shell‐enclosed kernel, although such compact structure seems to make oxygen permeation difficult. By using the SEM, we recently observed a continuous disintegration in different areca fruit tissues (including pericarp, mesocarp, and kernel membrane) with the prolongation of storage at ambient temperature (25°C) (Guo et al., 2020). In addition, some studies suggested that browning of the areca nut kernel could be possibly attributed to chilling injury (CI) (Li et al., 2016; Zhang, Li, et al., 2017). However, the mechanism involved in browning of areca nut kernel is not well understood yet. Therefore, the purpose of this study is to confirm the effects of storage at different temperatures, including those responsible for natural senescence and chilling injury, on kernel browning of the areca nut, and to investigate the possibility of oxygen entering the tissues from the perspective of morphological changes and physiological metabolism.

## 2 MATERIALS AND METHODS

### 2.1 Fruit material and treatments

Areca nuts (*A. catechu* L.) were collected at the mature green stage (pericarp hue angle of 117.23 ± 0.45 and single fruit weight of 30.85 ± 2.62 g, *n* = 10) from a commercial orchard located in Wanning city, Hainan Province, PR China. The harvested fruit was transported to the laboratory within 6 hr. After holding overnight at room temperature, fruit with uniformity of size and appearance, and without mechanical damage, disease or pests were chosen and divided into 3 groups, with 455 fruit for each group. The selected fruit was washed, placed in 300 mg/L prochloraz solution for sterilization at room temperature for 3 min, air‐dried, and then packed in polyvinyl chloride (PVC) plastic bags (200 mm ×150 mm), with 40 fruit for each bag. The 3 groups of packed fruit were transferred to 25 ± 0.5, 10 ± 0.5, and 5 ± 0.5°C, respectively, and stored for 20 d. During storage, the physiological and biochemical parameters were analyzed as described below.

### 2.2 Measurements of kernel browning index

The areca nuts were randomly selected and cut longitudinally to assess the kernel browning. The kernel browning index was evaluated by assessing the extent of l kernel browning symptoms using the following scales (Wang & Sugar, 2013): 0 = no browning; 1 = very slight browning; 2 = slight browning; 3 = moderate browning; and 4 = severe browning. The browning index was calculated using the formula: browning index (%) = ∑ (browning scale × number of fruit in each browning scale)/(total number of fruit investigated × 4) × 100. Each treatment had three replicates, with 15 fruit per treatment at each time point.

### 2.3 Determination of kernel shell membrane permeability

Membrane permeability of kernel shell was expressed by the relative leakage rate according to the method of Zhang, Zhu, et al. (2017). Ten kernel shell disks were derived from 5 fruit with a cork borer (10 mm in diameter), washed twice, and incubated in 30 ml of distilled water at 25°C for 30 min. Initial electrolyte value was determined with a conductivity meter (Model DDS‐307, INESA Analytical Instrument Co., Ltd, Shanghai, China). Then, the solution with disks was boiled for 15 min, quickly cooled to 25°C, and replenished with distilled water to 30 ml, and the total electrolyte value was again measured. Relative electrolyte leakage was calculated as a proportion (%) of initial electrolyte value to total electrolyte value. Each treatment contained three replicates, 5 fruit per replicate at each time point.

### 2.4 Assay of lignifications in mesocarp

The determination of lignin content was modified with reference to the method of Morrison et al. (1994). One gram of mesocarp tissue was added to 5 ml of precooled ethanol (95%, v/v) for grinding and then centrifuged at 12,000 × *g* for 10 min at 4°C. The precipitate was then centrifugally washed twice with a mixture of 5 ml precooled ethanol and n‐hexane (v/v = 1/2). The precipitate was collected, dried at 50°C, and then transferred to 1 ml of acetyl bromide glacial acetic acid (25%, v/v) solution. After heating in a water bath for 30 min at 70°C, 1 ml of 2 mol/L NaOH and 0.8 ml of 1 mol/L hydroxylamine hydrochloride were successively added to the reaction solution, followed by transfer to a 10‐mL volumetric flask, where the volume was adjusted with glacial acetic acid. Finally, the solution was centrifuged at 4,000 × *g* at 4°C for 10 min, and the absorbance of the supernatant was measured at 280 nm. The lignin content was expressed as mg/g fresh weight (FW).

Phenylalanine ammonia‐lyase (PAL) activity was analyzed by using a modified method of Assis et al. (2001). Two grams areca nut mesocarp was homogenized in 5 ml of precooled (4°C) sodium borate buffer (0.1 M, pH 8.8) containing 4% (m/v) polyvinylpyrrolidone, 5 mM β‐mercaptoethanol, and 2 mM EDTA. The homogenate was centrifuged at 12,000 × *g* for 20 min at 4°C. The supernatant was used as the crude enzyme extract. An enzyme extract (0.5 ml) was incubated with the reagent containing 3.5 ml of 0.05 M sodium borate buffers (pH 8.8) and 1 ml of 20 mM L‐phenylalanine as substrate at 40°C for 1 hr. The reaction was terminated by adding 0.2 ml of 6 M HCl. PAL activity was measured by change in absorbance at 290 nm. One unit of enzyme activity (U) was defined as the change in absorbance of 0.01 per hour per gram FW. PAL activity was expressed as U/g FW.

Each treatment for these two parameters contained three replicates, 5 fruit per replicate at each time point.

### 2.5 Assay of phenolic oxidation in kernel

Total phenolic content was measured according to Mishra et al. (2013) with slight modification. One gram of frozen areca nut kernel tissue was ground with 5 ml of precooled ethanol (95%, v/v) and then centrifuged at 12,000 × *g* for 20 min at 4°C. The supernatant was saved as phenolic compound extract. Ten‐fold diluted phenolic (0.1 ml) compounds extract was mixed with 1.9 ml of distilled water and 2 ml of Folin–Ciocalteu reagent. After shaking for 3 min, 2 ml of 10% (m/v) Na_2_CO_3_ solution was added. The absorbance of mixture was measured at 760 nm after reacting for 60 min. The phenolic content was expressed as milligram gallic acid equivalents (GAE) per gram FW.

Polyphenol oxidase (PPO) activity was measured by the method of Li (2000) with minor modifications. Two grams areca nut kernel and 5 ml phosphate buffer solution (pH5.8) were ground into homogenate in ice bath and then centrifuged at 12,000 × g for 20 min. The supernatant (0.5 ml) was incubated with assay medium containing 3.5 ml of 0.05 M PBS (pH 7.0) and 1 ml of 0.05 M catechol as substrate for 30 s, and the absorbance was measured at 420 nm. One U of PPO activity was defined as the amount of enzyme causing 0.001 absorbance increase per minute at 420 nm. PPO activity was expressed as U/g FW.

Each treatment for both parameters contained three replicates, 5 fruit per replicate at each time point.

### 2.6 The ultrastructural observation of scanning electron microscopy (SEM)

For SEM observation, the different areca nut tissues including pericarp, mesocarp, and kernel shell were separately sampled from 5 fruit per treatment at 20 d of storage, each part of tissues in each fruit was cut into 2‐mm pieces and immediately put into 2.5% glutaraldehyde solution at pH 6.8. Following fixation at 4°C for 24 hr, they were rinsed with 0.1 mol/L phosphate buffer (pH 7.2) and dehydrated in 50%, 70%, 80%, 90%, and 95% ethanol for 15 min and 100% ethanol for 30 min. After 48 hr of vacuum freeze‐drying, the samples were pasted on the sample table with conductance adhesive, and the gold‐plated films of the ion sputtering coating apparatus were placed under an SEM (S‐3000N, J Hitachi Ltd., Tokyo, Japan) at 10 kV acceleration to observe and obtain images.

### 2.7 Statistical analysis

The experiment was arranged on the basis of completely randomized design with three replications. Data in the figures are presented as the means ± standard errors (SE). One‐way analysis of variance (ANOVA) test was performed, and means at same sampling day were compared by Duncan's multiple range tests at *p* = .05 using statistical software (SPSS version 19.0; Chicago, IL).

## 3 RESULTS

### 3.1 Browning index of the areca nut kernel

**FIGURE 1:**
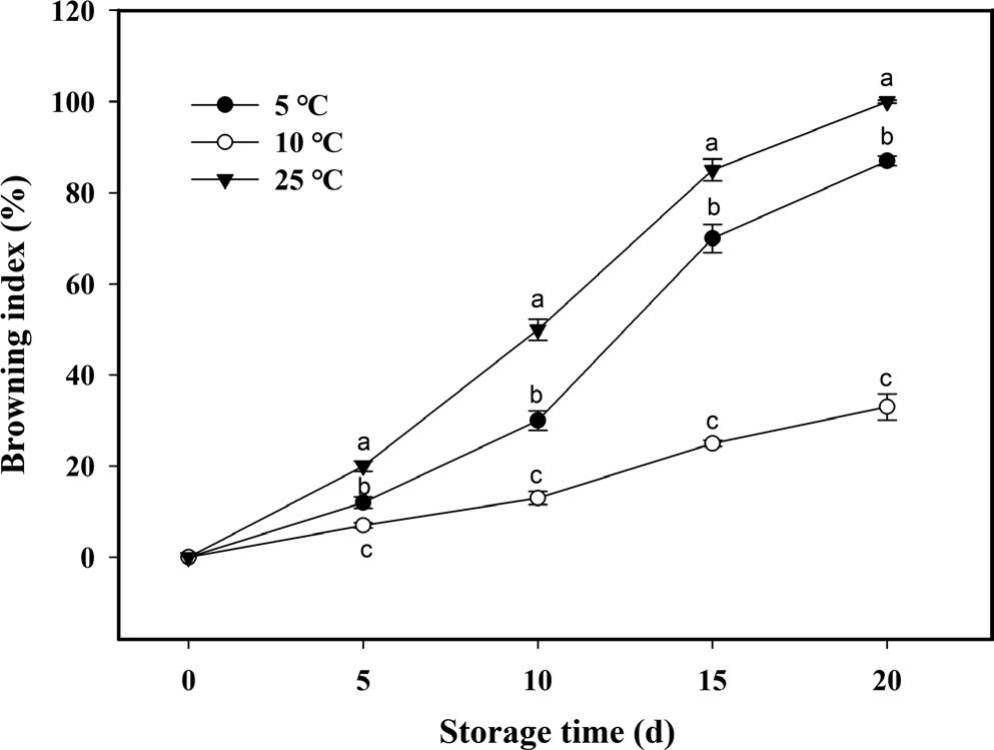
Changes of browning index in areca nut kernel (B) at 5, 10, and 25°C. Data are expressed as mean of triplicate samples. Vertical bars represent the standard errors of the means. Different letters at the same time point are significantly different (*p* < .05)

The kernel browning indexes in areca nuts stored at 5, 10, and 25°C showed increasing trends with different degrees (Figure 1). The most rapid increase in the kernel browning index was found in areca nuts stored at 25°C, in which the browning index reached to 100% after 20 d of storage (Figure 1). Kernel browning severity in areca nuts stored at 5°C was in the second place, with browning index reaching to 87% (Figure 1). By contrast, areca nuts at 10°C showed the lowest browning severity, with the index increasing to 33% at the end of storage (Figure 1).

### 3.2 Membrane permeability of kernel shell

**FIGURE 2:**
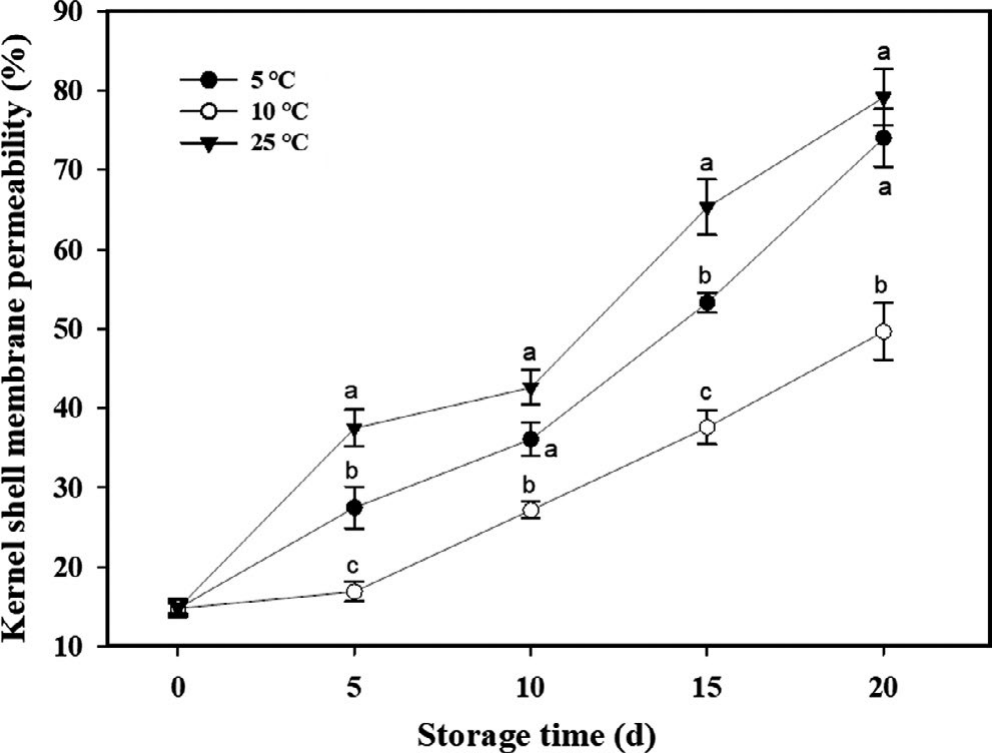
Changes of membrane permeability in areca nut kernel shell at 5, 10, and 25°C. Data are expressed as mean of triplicate samples. Vertical bars represent the standard errors of the means. Different letters at the same time point are significantly different (*p* < .05)

Similar to changes in kernel browning indices, the kernel shell membrane permeability in areca nuts continuously increased during storage (Figure 2). The increasing rate of membrane permeability was affected by different storage temperatures, with ranking being 25°C > 5°C > 10 ºC (Figure 2).

### 3.3 Mesocarp lignin content and PAL activity

**FIGURE 3:**
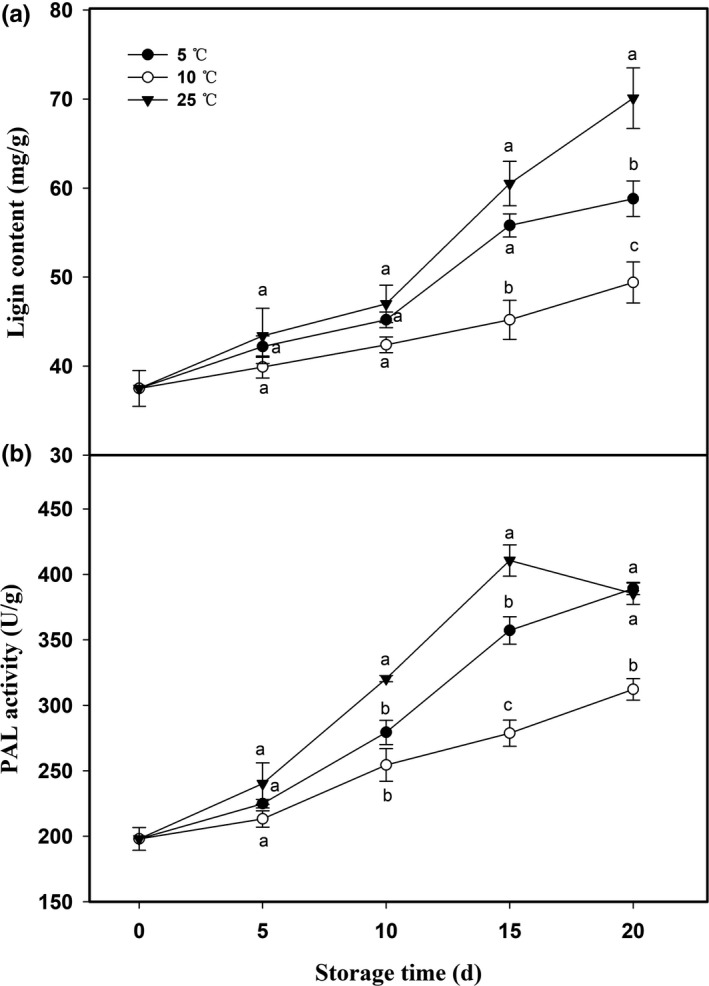
Changes of lignin content (a) and PAL activity (b) in areca nut mesocarp at 5, 10, and 25°C. Data are expressed as mean of triplicate samples standard errors. Vertical bars represent the standard errors of the means. Different letters at the same time point are significantly different (*p* < .05)

Areca nuts showed different degrees of increase in mesocarp lignin contents throughout storage at 25, 10, and 5°C (Figure 3a). Areca nuts stored 25 and 5°C showed 1.87‐fold and 1.57‐fold increases in lignin contents over the storage, respectively (Figure 3a). Comparatively, less increase in lignin content was observed in mesocarp of areca nuts stored at 10°C.

PAL is a key enzyme in the phenylpropanoid pathway, which is responsible for synthesis of phenolics and lignin. The PAL activity in areca nut mesocarp at 25°C steadily increased and reached its peak at 15 days, at which the PAL activity was 2.1 times as much as initial activity, followed by a slight decrease (Figure 3b). In general, PAL activity in areca nuts stored at 25°C was the highest among three storage temperatures, with the exception of 20 days (Figure 3b). PAL activity in areca nuts stored at 5 and 10°C constantly increased, but areca nuts at 10°C showed a lower PAL activity than that at 5°C (Figure 3b).

### 3.4 Total phenolic content and PPO activity in kernels

**FIGURE 4:**
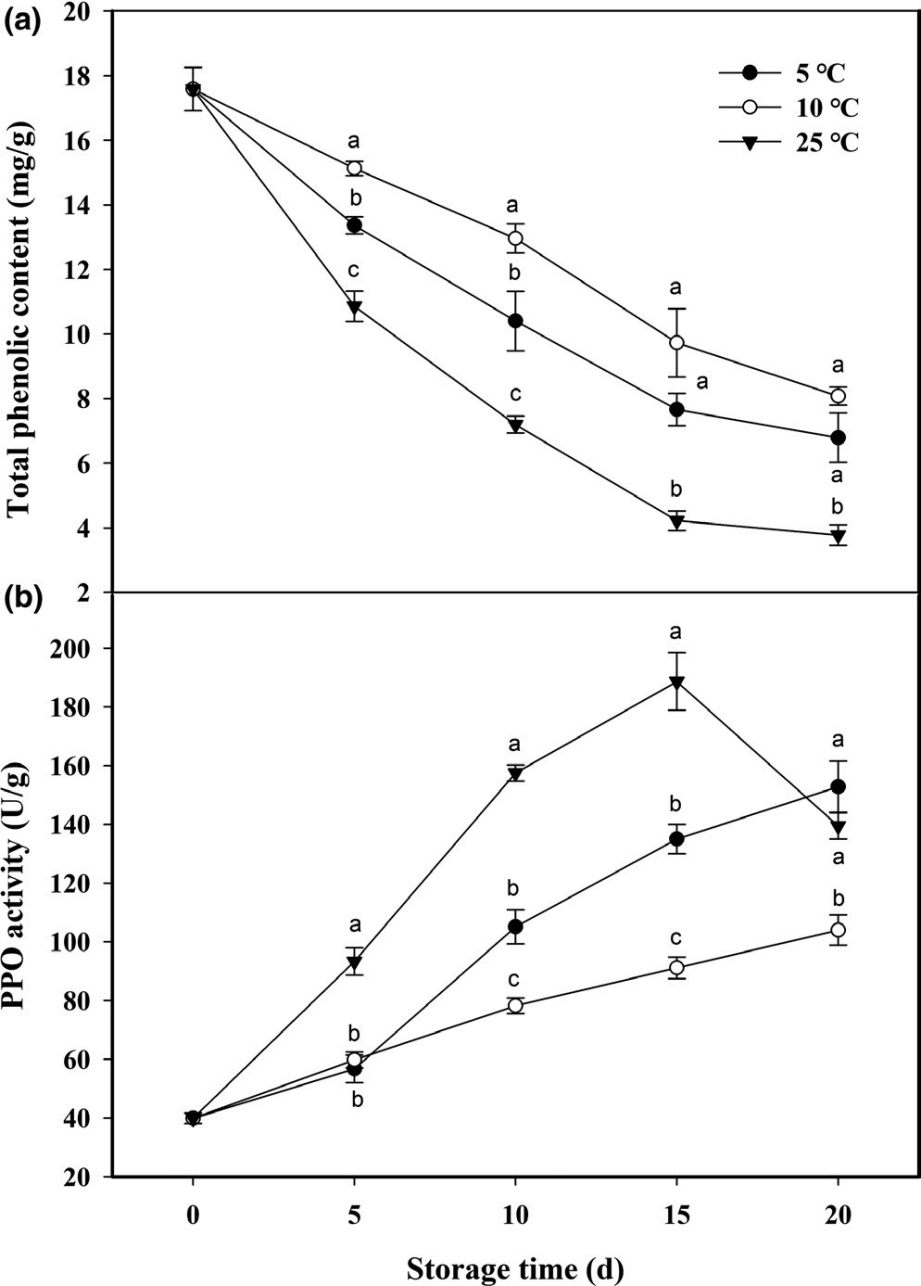
Changes of total phenolic content (a) and PPO activity (b) in areca nut kernel at 5, 10, and 25°C. Data are expressed as mean of triplicate samples standard errors. Vertical bars represent the standard errors of the means. Different letters at the same time point are significantly different (*p* < .05)

Total phenolic content in areca nut kernel of all treatment groups showed steady declines during storage (Figure 4a). Differently, total phenolic content in kernel of areca nut stored at 25°C showed the fastest decreasing rate, followed by that at 5°C, while the slowest decreasing rate was observed in kernel of areca nut stored at 10°C (Figure 4a). PPO activity in areca nut kernel rapidly increased and reached a maximum at 15 days of storage at 25°C, followed by a sharp decrease (Figure 4b). Low temperatures (5 and 10°C) storage suppressed the increase in PPO activity, to a certain degree (Figure 4b). Comparatively, PPO activity in areca nut kernel stored at 5°C was overall higher than that in kernel at 10°C, with the exception of 5 days of storage (Figure 4b).

### 3.5 Changes in the ultrastructure of the areca nuts

#### 3.5.1 The ultrastructure of pericarp

**FIGURE 5:**
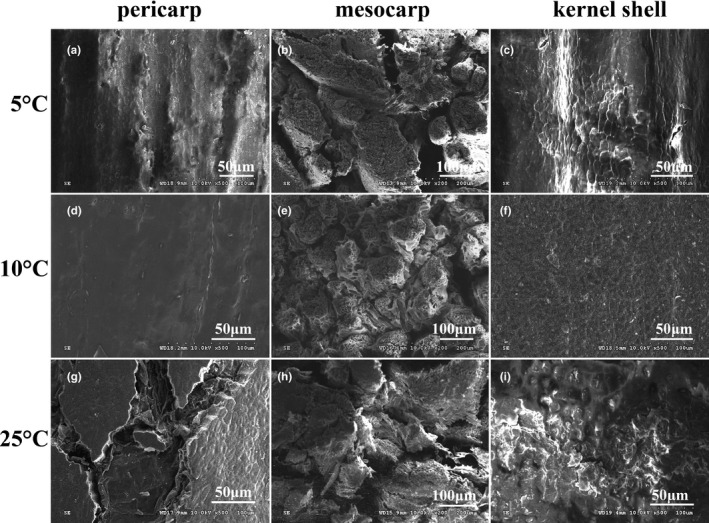
Scanning electron micrographs of pericarp, mesocarp, and kernel shell in areca nut at 5°C and 10°C. a–c, pericarp, mesocarp, and kernel shell structure of areca nuts stored at 5°C for 20 days; d–f, pericarp, mesocarp, and kernel shell structure of areca nuts stored at 5°C for 20 days; g–i, pericarp, mesocarp, and kernel shell structure of areca nuts stored at 5°C for 20 days. Figures g, h, and i were cited and reprinted from our previous reference (Guo et al., 2020; Food Sci. Nur. 8, 1818‐1827)

The epidermal structure of the areca fruit at the initial stage of storage (0 day) was relatively complete, with a tight and waxy surface coverage, no fragments, and a short crack, as depicted in our previously report (Guo et al., 2020). Occurrence of chilling injury in areca nuts at 5°C led to aggravated deterioration of pericarp tissue, in which the cutin membrane of the areca nut pericarp showed silk texture, holes, naked scales, and an increase in the length and width of furrows (Figure 5a). Areca nuts stored at 10°C were covered with wax throughout the storage and maintained a relatively stable structure (Figure 5d). Comparatively, pericarp cells of areca nuts at 25°C suffered the most serious damage, in which the wax decomposed gradually and dispersed discontinuously, with showing obvious fragmentation and indentation into the lenticular cells (Figure 5g). Additionally, the cuticle of the pericarp of areca nut stored at 25 ºC was partially exfoliated, exposed in a honeycomb shape, and was split on the surface to form lenticels (Figure 5g). Obviously, the destruction of the pericarp tissue in areca nuts stored at 25 and 5°C would facilitate the ingress of oxygen.

#### 3.5.2 The ultrastructure of mesocarp

At the initial stage of storage, the mesocarp tissue of areca nut arranged in a neat, orderly, and tight manner with small cell space (Guo et al., 2020). After storage of 20 days at 5°C, the mesocarp tissue structure of the areca fruit became disordered, in which the connections between tissues relaxed, mesocarp tissue separated from others and resulted in increased mesocarp tissue space (Figure 5b). The degree of tissue integrity of mesocarp tissue of areca nut at 20 days of storage at 25°C (Figure 5h) was similar to that of areca nut stored at 5°C (Figure 5b). Comparatively, a slight structural change with small cell space was observed in mesocarp tissue of areca nut after 20 days of storage at 10°C (Figure 5e).

#### 3.5.3 The ultrastructure of the areca nut kernel shell

The initial stage of storage, the kernel shell structure was intact and smooth (Guo et al., 2020). After 20 days of cold storage at 5°C, the cracks appeared at cell membrane in kernel shell of areca nut (Figure 5c). The damage to the kernel shell was more severe in areca nut stored at 25°C on 20 days (Figure 5i). Differently, after 20 days of storage at 10°C, the structural change in the areca nut kernel shell was relatively minimal, and the integrity of areca nut kernel membrane was well maintained (Figure 5f).

## 4 DISCUSSION

This study clearly showed that areca nut kernel was susceptible to postharvest browning during natural senescence at 25°C and chilling stress at 5°C, and the browning symptom was more severe at 25°C than that at 5°C. By contrast, a delayed browning development was noted in areca nut when exposed to a milder low temperature condition (10°C). Hereby, we propose that chilling stress may aggravate the browning of the areca nut, but natural senescence may be more important factor leading to browning. Similar observations were demonstrated in “Whangkeumbae” pear fruit (Wang et al., 2008). Be somewhat differently from pineapple, in which low temperature is generally proposed as the main postharvest factor that causes black heart disease (Ko et al., 2013; Zhou et al., 2003).

Under normal circumstances, the oxygen entering the fruit tissue is extremely limited due to layers of barriers from the pericarp, lenticels, and other normal channels, which can avoid the occurrence of internal browning in many postharvest fruits (Sun et al., 2002). Therefore, a possible reason for internal enzymatic browning of areca nut could be due to change in the structure of the fruit tissues, which might facilitate the entry of oxygen into interior tissues. The present results observed by SEM showed that different parts of the areca nut tissue had significant structural changes at varying storage temperatures. The pericarp is the outermost layer of vegetable tissue, and the most striking feature of pericarp cells is to possess the thick outer wall. The pericarp is covered by a layer of cuticle that is composed of two major components including cutin and cuticular waxes (Benítez et al., 2019; Zhao et al., 2019). Plant cuticles perform diverse biological functions, such as regulation of gas exchange, interaction with pathogens and insects, and protection against different abiotic and biotic stresses (Heredia‐Guerrero et al., 2018). The present results by SEM observations showed that the coverage of wax on the areca nut skin decomposed gradually with the extension of storage time. Once lenticels are formed, they become the main portal for gas entry instead of pores (Groh et al., 2002). This structural change would cause increases in skin permeability, thereby greatly increasing the opportunity of gas exchange and water evaporation. Similarly, during the development of black heart disease in “Ya” pear fruit, the wax of the fruit pericarp decreased gradually, the wax layer became thinner, the pericarp cleft became wider and longer, and the cells at the lenticels were exposed (Huo & Li, 1992). In “Nanguo” pear, in addition to decreased epidermal wax, the cuticle showed large‐scale exfoliation while the dead pericarp cells after bolting were exposed, resulting in obvious cellular disorder (Li et al., 2018). Our study showed that increasing kernel shell membrane permeability at 25 and 5°C was tightly correlated with disintegration of pericarp structure. Consequently, oxygen might more easily permeate into tissues and cells. As oxygen passes through the pericarp, it would enter the mesocarp.

In case of lignification in mesocarp tissue, the blocking effect on permeability of external gasses may be greatly impaired because lignified cells are dead cells (Singh et al., 2010). With the development of mesocarp lignification of the areca nut fruit, the arrangement of mesocarp tissues became disordered, the connection between tissues loosened gradually, the connected parts reduced gradually, and the separation from other tissues led to an increase in mesocarp tissue space (Guo et al., 2020). The present results demonstrated that the mesocarp tissue of the areca nut was lignified during natural senescence and chilling stress, as indicated by higher lignin content at 25 and 5°C, which might increase oxygen infiltration into interior tissue. Studies on loquats (Cai et al., 2006), mangoes (Dangcham et al., 2008), eggplants (Xie et al., 2016), and kiwifruits (Suo et al., 2018) revealed that ripening and chilling injury could promote fruit lignification, which was consistent with the results obtained from the present study. In addition to lignification, the senescence and chilling injury of the fruit also accelerated the destruction of mesocarp tissue (Rao & Ren, 1997). Compared to 10 ℃ in this study, more evident morphological changes were observed in the mesocarp tissue of the areca nut at 25 and 5 ℃, as shown by gradual loose and separation in parallel with increased lignification and widened tissue gaps, which might increase the penetration of oxygen into the mesocarp and accelerate the enzymatic browning process.

Unlike pineapple, pear, and other fruits whose internal browning occur in the mesocarp tissue, browning of areca nuts appeared in the kernel. In the present study, higher kernel shell membrane permeability was observed in areca fruit stored at 25 and 5°C, indicating that natural senescence and severe chilling stress might trigger greater damage to kernel shell structure, which could make oxygen more accessible to areca nut kernel and initiate enzymatic browning. Furthermore, the SEM observations showed that the kernel shell of areca fruit stored at 25 and 5°C had both shrinks and cracks that were aggravated with the extension of storage. The changes in kernel shell structure could make oxygen more accessible to areca kernel and initiate enzymatic browning. The viewpoint can be consolidated by further results, in which higher PPO activity in relation to more severe browning was detected in areca kernel during storage at 25 and 5°C, implying that adequate oxygen existed in areca kernel under these temperatures. Comparatively, a moderately low temperature (10°C) might be helpful in slowing the appearance of fission in the shell structure and reducing the occurrence of kernel browning.

## 5 CONCLUSION

Natural senescence under ambient temperature (25°C) was a main factor that induced kernel browning of the areca nut, while the occurrence of chilling injury under severe chilling stress might also aggravate kernel browning of the areca nut. During senescence or exposure to severe chilling stress, the areca nut tissues were vulnerable to damage or lignification, which could allow the ingress of excessive oxygen that exceeds the demands for normal physiological metabolism in areca nut kernel. In addition, both temperatures (25 and 5°C) induced the increase in PPO activity in kernel and therefore leading to enzymatic browning. Moderate low temperature (10°C) might help maintain tissue integrity, which could be conducive to reducing the occurrence of areca kernel browning.

## CONFLICT OF INTEREST

The authors declared that they have no conflict of interest.

## ETHICAL APPROVAL

This study does not involve any human or animal testing.

## Data Availability

The research data are not shared.
